# Classification Based on Pruning and Double Covered Rule Sets for the Internet of Things Applications

**DOI:** 10.1155/2014/984375

**Published:** 2014-01-05

**Authors:** Shasha Li, Zhongmei Zhou, Weiping Wang

**Affiliations:** Department of Computer Science and Engineering, Minnan Normal University, Zhangzhou 363000, China

## Abstract

The Internet of things (IOT) is a hot issue in recent years. It accumulates large amounts of data by IOT users, which is a great challenge to mining useful knowledge from IOT. Classification is an effective strategy which can predict the need of users in IOT. However, many traditional rule-based classifiers cannot guarantee that all instances can be covered by at least two classification rules. Thus, these algorithms cannot achieve high accuracy in some datasets. In this paper, we propose a new rule-based classification, CDCR-P (Classification based on the Pruning and Double Covered Rule sets). CDCR-P can induce two different rule sets *A* and *B*. Every instance in training set can be covered by at least one rule not only in rule set *A*, but also in rule set *B*. In order to improve the quality of rule set *B*, we take measure to prune the length of rules in rule set *B*. Our experimental results indicate that, CDCR-P not only is feasible, but also it can achieve high accuracy.

## 1. Introduction

The Internet of things is one of the hot topics in recent years. It has integrated many kinds of modern technology. By these kinds of technology, it produces large-scale data in IOT. In order to handle these large data, it requires techniques and methods of data mining and machine learning [1–6].

As one of the most important tasks of data mining, classification has been widely applied in IOT. The main idea of classification is that builds classification rules. According these rules, we can predict the class label for unknown objects.

Traditional rule-based classifications usually use greedy approach, such as FOIL [7], CPAR [8], and CMER [9]. These methods repeatedly search for the current best one rule or best-*k* rules and remove examples covered by the rules. They cannot guarantee that all instances can be covered by at least two classification rules. As a result, some traditional classifiers have less classification rules. Their accuracy may not be high. Decision tree classifiers produce classification rules by constructing classification trees, such as ID3 [10], C4.5 [11], and TASC [12]. The process of building a decision tree does not need to delete any examples. All examples can find only one matching rule in the classification rule set. That is why decision trees often generate small rule sets and cannot achieve high accuracy in some data.

Aiming at these weaknesses, we propose a novel Double Covered Rule sets classifier called CDCR-P (Classification based on the Pruning and Double Covered Rule sets). CDCR-P generates two different rule sets *A* and *B* and then prunes the rule set *B*. Each instance can be covered by at least one rule from rule set *A*. At the same time, each instance can be covered by at least one rule from rule set *B*. CDCR-P has four aspects. First, CDCR-P generates rule set *A*. We select several best values which can just cover the training set to construct a candidate set. CDCR-P employs candidate set to produce rule set *A*. Second, in order to induce rule set *B*, we remove the values of candidate set in training data and select other several best values to induce rule set *B*. Rule set *A* is fully different from rule set *B*. Third, each instance can find at least two matching rules. One of the rules is from rule set *A*, and another is from rule set *B*. Forth, we prune the length of rules in rule set *B*, so as to improve the quality of rule set *B*. Our method has the following advantages.

1. CDCR-P can produce two rule sets. Thus, CDCR-P can generate large number of classification rules.
2. All instances in training set can be matched by at least two classification rules.
3. CDCR-P can achieve high accuracy by combining rule set *A* with rule set *B*.

The paper is organized as follows. In Section 2, we introduce the method of CVCR (Classification based on Value Covered Rules). In Section 3, we propose a new classifier CDCR-P and discuss how to use CDCR-P to classify new objects. We report our experimental results in Section 4. We finally conclude our study in Section 5.

## 2. Classification Based on Value Covered Rules

In this section, we introduce the method of value covered classifier; this method is called CVCR (Classification based on Value Covered Rules).

Suppose *T* = {*t*_1_, *t*_2_,…, *t*_*n*_} is a set of tuples. Each tuple *t* has *m* attributes {*A*_1_, *A*_2_,…, *A*_*m*_}. Let *C* be a finite set of class labels {*C*_1_, *C*_2_,…, *C*_*k*_} and *S* be a set consisting of *s* data samples. A rule *r* consists of several samples *s* and a class label *c*, which takes the form of *s*_1_∧*s*_2_∧⋯∧*s*_*l*_ → *c*. One rule set is formed by a lot of rules which are extracted from one classifier. If tuple *t* satisfies *s*_1_∧*s*_2_∧⋯∧*s*_*l*_ from rule *r*, the *t* is matched by *r*. *r* predicts that *t* belongs to class *c*.

**Definition 1 (information gain)**

Let *s*_*i*_ be the number of samples of *S* in class *C*_*i*_. The information gain of an attribute value is denoted by *I* (*s*_1_, *s*_2_,…, *s*_*k*_) and is defined as follows:

(1) $$\begin{array}{c} I\left(s_{1},s_{2},\ldots ,s_{k}\right)=-\sum_{i=1}^{k}p_{i}\;\text{lo}{\text{g}}_{2}\left(p_{i}\right), \end{array}$$

where *p*_*i*_ is the probability that a literal belongs to class label *c*_*i*_. *p*_*i*_ is estimated by *s*_*i*_/*s*.

CVCR finds a set of values which can cover all the training set. The process of constructing CVCR is as follows.

First, CVCR sorts all literals according to the information gain in a descending order and selects several best attribute values *v*_1_, *v*_2_,…, *v*_*i*_ which can just cover the training set *T*. *v*_1_, *v*_2_,…, *v*_*i*_ construct a candidate set. Let these values split *T* to subdatasets *t*_1_, *t*_2_,…, *t*_*i*_, respectively. Second, CVCR connects *v*_*i*_ with attribute values *v*_*i*1_, *v*_*i*2_,…, *v*_*ij*_ which can just cover dataset *t*_*i*_ to produce patterns. Finally, repeat the above steps until the information gain of each pattern is equal to 0.

The experimental results of CVCR are shown in Table 2. The experimental results show that CVCR can achieve higher accuracy than ID3 and FOIL. Because CVCR contains the global optimal attribute values, CVCR is more feasible than ID3. However, CVCR still produces less classification rules, which cannot guarantee that each instance can be matched by at least two rules.

## 3. Classification Based on Pruning and Double Covered Rule Sets

In this section, we produce a new method CDCR-P. First, we show the process of how to induce rule sets *A* and *B*. Second, we describe the method of how to prune rule set *B*. Finally, we give the way of how to use the two rule sets *A* and *B* to classify new objects.

### 3.1. Constructing Rule Sets *A* and *B*

Based on the idea of CVCR, we continue mining knowledge in-depth. This approach divides the training set *T* into three small datasets *T*_1_, *T*_2_,  and  *T*_3_ according to candidate set. The method contains four steps. Step 1, we select several best attribute values *v*_1_, *v*_2_,…, *v*_*k*_ from candidate set which can just cover one-third of training set. Attribute values *v*_1_, *v*_2_,…, *v*_*k*_ have less information gain. The tuples which contain one of *v*_1_, *v*_2_,…, *v*_*k*_ form the small dataset *T*_1_. The process is shown as Algorithm 1. We form *T*_2_, *T*_3_ using the same way as *T*_1_. Step 2, according to *v*_1_, *v*_2_,…, *v*_*k*_,  *T*_1_ is split into datasets *t*_1_, *t*_2_,…, *t*_*n*_. We find cv_*i*_ (a set of cover values) from *t*_*i*_ on the basis of information gain. The measure of cv_*i*_ is the same as CVCR, shown as Algorithm 2. CDCR connects *v*_*i*_ with cv_i_ to produce patterns. If the information gain of pattern is equal to 0, *X* → *C* belongs to rule set *A*, shown as Algorithm 3. Step 3, CDCR recalculates the CV (cover values) in *T*_1_ excluding *v*_1_, *v*_2_,…, *v*_*k*_. CV splits *T*_1_ into some datasets and connects covered value in each dataset to produce new rules. These rules belong to rule set *B*, shown as Algorithm 4. Finally, we remove *T*_1_ from *T* and iterate the process until *T*_2_, *T*_3_ are trained. Rule set *A* is the same as CVCR. Both rule sets *A* and *B* belong to CDCR.

### 3.2. Pruning Rule Set *B*

In order to improve the quality of rule set *B*, we introduce a new method CDCR-P (Classification based on the Pruning and Double Covered Rule sets).

**Definition 2 (confidence)**

The confidence of sample *X* is defined as follows:

(2) $$\begin{array}{c} \text{conf}\left(X\right)=\frac{\text{count}\left(X_{c}\right)}{\text{count}\left(X\right)}\times 100\%, \end{array}$$

where count (*X*_*c*_) means the number of tuples which contain sample *X* in class *c*.

The confidence of rules that CDCR generated is equal to 100%. We modify the length of rule set *B*. The rules are generated when the confidence is 100% in the small dataset *T*_*i*_ instead of in the whole training set *T*. Each rule is marked with the confidence in *T*. Thus, rule set *B* in CDCR-P is shorter than rule set *B* in CDCR.

### 3.3. Classifying Unknown Examples

In this part, we give the method of how to use CDCR and CDCR-P to classify unknown instances.

**Definition 3 (support)**

The support of sample *X* is denoted by

(3) $$\begin{array}{c} \sup\left(X\right)=\frac{\text{count}\left(X\right)}{\left|T\right|}\times 100\%, \end{array}$$

where count(*X*) means the number of tuples which contain sample *X*. |*T*| is the number of tuples in training data.

When testing unknown examples, CDCR selects the matched rule with the highest support. If some rules have the same support, we select the maximum number of matched rules in each class.

CDCR-P first considers the rule with the highest confidence. If two rules have the same confidence, CDCR-P sorts the two rules according to the support.

**Definition 4 (missing match rate)**

If the test instance cannot find any match rule, this unclassified instance is considered mismatch. The missing match rate is defined as

(4) $$\begin{array}{c} \frac{\text{count}\left(\text{unclassified}\;\text{instance}\right)}{\left|T\right|}\times 100\%, \end{array}$$

where count (unclassified instance) means the number of tuples which cannot be matched by rules.

## 4. Experiments

We show the experimental results in 14 UCI datasets. The character of each data is shown in Table 1. All the experiments are performed on a 2.2 GHz PC with 2.84 G main memory, running Microsoft Windows XP. Experiments run tenfold cross validation method for each data.

**Figure 1:**
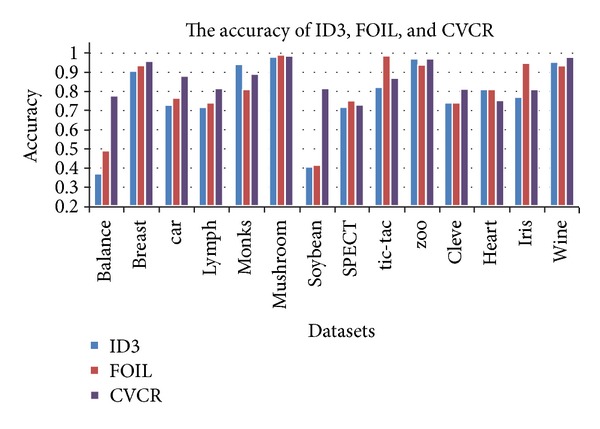
The accuracy of ID3, FOIL, and CVCR.

**Figure 2:**
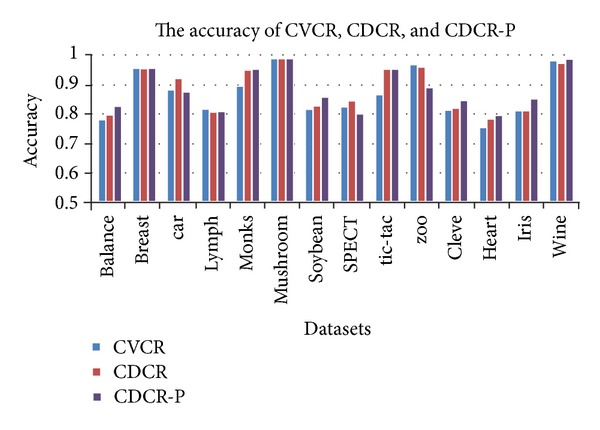
The accuracy of CVCR, CDCR, and CDCR-P.

In Table 2, we give the accuracy of ID3, FOIL, CVCR, CDCR, and CDCR-P.  Figure 1 gives the accuracy of ID3, FOIL, and CVCR. CVCR employs the idea of covered values; these cover values are the global optimal attribute values in training data. From Figure 1 and Table 2 we can see that CVCR can achieve higher accuracy than ID3 and FOIL.  Figure 2 gives the accuracy of CVCR, CDCR, and CDCR-P. CDCR not only uses the method of covered values, but also produces two rule sets *A* and *B*. Each instance can be matched at least by one rule from rule set *A* and rule set *B*. From Figure 2 and Table 2 we can see that CDCR can achieve higher accuracy than CVCR. Based on all advantages of CVCR and CDCR, CDCR-P take measure to prune the length of rule set *B*. The experimental results show that CDCR-P has the highest accuracy.

Table 3 displays the missing match rate of ID3, FOIL, CVCR, CDCR, and CDCR-P. CVCR can produce more rules than ID3 and FOIL. From Table 3 we can see that the missing match rate is decreased obviously by CVCR. CDCR produces two rule sets. Therefore, CDCR produces more rules than CVCR. From Table 3 we can see that the missing match rate of CDCR is lower than CVCR. CDCR-P modifies the length of rule set *B*; the quality of rules in CDCR-P is higher than CDCR. The experiments indicate that the mismatch rate of CDCR-P is the lowest.

Through all the above experimental results, we can conclude the following. (1) It is necessary for us to construct two rule sets. (2) It is necessary to prune rule set *B*. (3) CDCR-P can achieve high accuracy and has an excellent result in missing match rate.

## 5. Conclusions

Classification has been widely applied in IOT. The accuracy of classification is an important factor in classification task. The traditional rule-based classifications cannot guarantee that all test cases can be matched by two rules. They usually generate less classification rules. Thus, the accuracy of these algorithms may be low in some data. In this paper, a novel approach CDCR-P is proposed. CDCR-P generates two rule sets: rule set *A* and rule set *B*. All instances can be matched by at least one rule not only in rule set *A*, but also in rule set *B*. This method greatly increases the number of extracted rules. Thus, it gets more information from training data. Our experimental results show that the methods of CDCR-P can produce more rules and achieve high accuracy. In future research, we will perform an in-depth study on combining distributed data mining with IOT in order to improve the efficiency of CDCR-P.

## Figures and Tables

**Algorithm 1:**
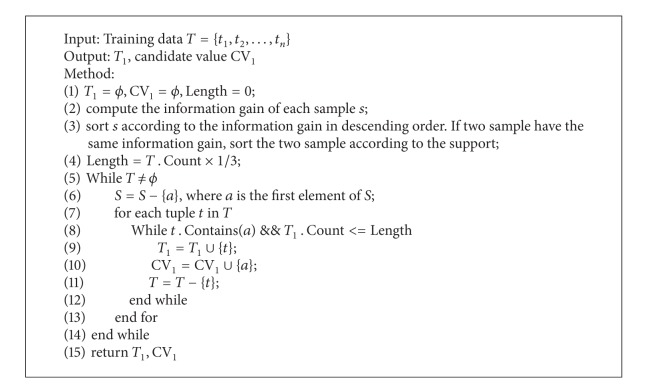
Dividing training set into three smalldatasets.

**Algorithm 2:**
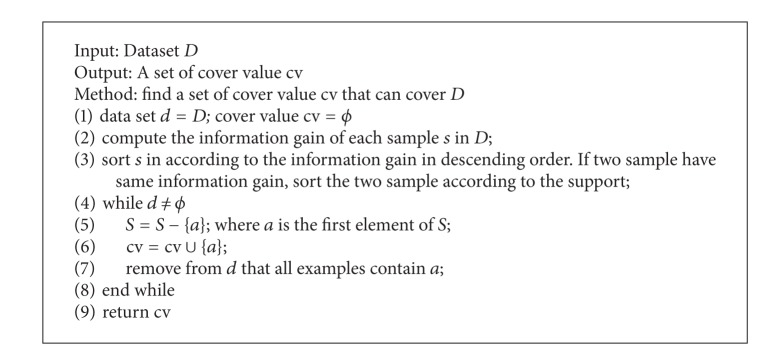
Finding covered values in dataset.

**Algorithm 3:**
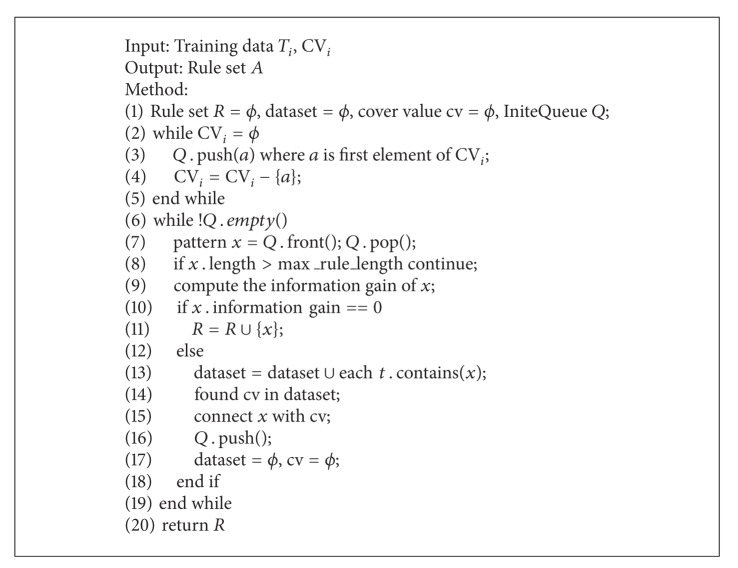
Inducing rule set A.

**Algorithm 4:**
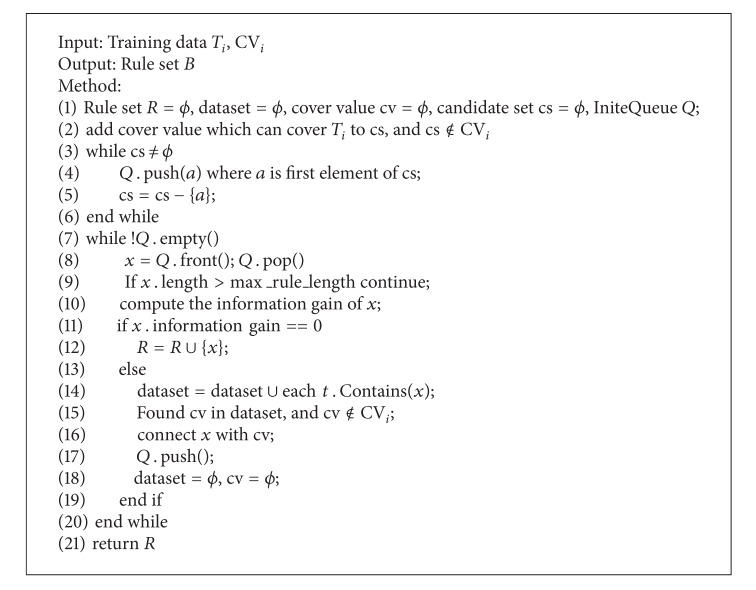
Inducing rule set *B*.

**Table 1:** Characteristics of UCI datasets.

Dataset	No. of instances	No. of attributes	No. of classes
Balance	625	5	3
Breast	699	10	2
Car	1728	7	4
Lymph	148	18	4
Monks	432	7	2
Mushroom	400	23	2
Soybean	307	36	19
SPECT	267	23	2
Tic-tac	958	10	2
Zoo	101	16	7
Cleve	303	13	2
Heart	270	13	2
Iris	150	4	3
Wine	178	14	3

**Table 2:** The accuracy of ID3, FOIL, CVCR, CDCR, and CDCR-P.

Dataset	ID3	FOIL	CVCR	CDCR	CDCR-P
Balance	0.3716	0.4929	0.7810	0.7985	0.8289
Breast	0.9042	0.9342	0.9571	0.9556	0.9585
Car	0.7298	0.7714	0.8837	0.9248	0.8767
Lymph	0.7148	0.7424	0.8181	0.81	0.81
Monks	0.9448	0.8146	0.8959	0.9514	0.9537
Mushroom	0.985	0.995	0.99	0.99	0.99
Soybean	0.4102	0.4172	0.8180	0.8276	0.8601
SPECT	0.7181	0.752	0.7303	0.7453	0.8053
Tic-tac	0.8215	0.9875	0.8684	0.9541	0.9530
Zoo	0.97	0.9409	0.9709	0.9609	0.8918
Cleve	0.7426	0.7423	0.8152	0.8216	0.8482
Heart	0.8148	0.8148	0.7556	0.7852	0.7963
Iris	0.7733	0.9533	0.8133	0.8133	0.8533
Wine	0.96	0.9379	0.983	0.9712	0.9882
Average	0.7758	0.8069	0.8629	0.8793	0.8867

**Table 3:** The missing match rate of ID3, FOIL, CVCR, CDCR, and CDCR-P.

Dataset	ID3	FOIL	CVCR	CDCR	CDCR-P
Balance	0.422	0.3518	0.0910	0.0799	0.0016
Breast	0.0529	0.0443	0.0029	0	0
Car	0.2165	0.1945	0.0231	0.0156	0
Lymph	0.0624	0.1014	0.0267	0	0
Monks	0	0.1023	0.0486	0.0486	0
Mushroom	0.005	0.005	0	0	0
Soybean	0.0559	0.2787	0.0097	0	0
SPECT	0.0566	0.0634	0.1387	0.1313	0
Tic-tac	0.0366	0.0094	0.0052	0	0
Zoo	0.01	0.0591	0	0	0
Cleve	0.0231	0.0795	0.0298	0.0265	0
Heart	0	0.0148	0.0704	0.0444	0.0111
Iris	0.1733	0.0067	0.1533	0.1533	0.06
Wine	0	0.0454	0.0056	0	0
Average	0.0796	0.0969	0.0432	0.0357	0.0052
